# Protective effect of Salvianolic acid B against atherosclerosis: a preclinical systematic review and meta-analysis

**DOI:** 10.3389/fphar.2025.1548811

**Published:** 2025-06-18

**Authors:** Xing Ji, Kailin Huang, Aimei Lu, Xiaomeng Hu, Huanlin Wu

**Affiliations:** ^1^ Dongzhimen Hospital, Beijing University of Chinese Medicine, Beijing, China; ^2^ Graduate School, Beijing University of Chinese Medicine, Beijing, China

**Keywords:** animal model, Salvianolic acid B (Sal B), atherosclerosis, meta-analysis, preclinical studies

## Abstract

**Background:**

Atherosclerosis is the most common cause of cardiovascular disease, with high morbidity and mortality rates globally. Salvianolic acid B (Sal B), the most active and abundant component of the water-soluble extract of the traditional Chinese medicine Danshen, has been demonstrated to exert atheroprotective effects; nonetheless, its protective potential remains unclear. This study aimed to evaluate the preclinical efficacy of Sal B in the treatment of atherosclerosis and summarize the relevant mechanisms of action to provide evidence for its use in the treatment of atherosclerosis.

**Methods:**

A systematic search was conducted across eight databases, including PubMed, Embase, and Web of Science, etc., for studies related to Sal B in animal models of atherosclerosis, published from the inception of these databases up to November 2024. Parameters such as atherosclerotic lesion area, lipid deposition, plaque size, lipid metabolism, and changes in inflammatory markers were used to assess the extent of the atherosclerotic lesions. The SYRCLE risk-of-bias tool was used to determine methodological quality. Data were analyzed using the STATA software. Time-dose effect analysis was performed to explore the relationship between Sal B and atherosclerosis.

**Results:**

Eleven studies involving 275 animals were analyzed. The results of these studies indicate that Sal B has a significant positive impact on various indicators of atherosclerosis. A meta-analysis of preclinical studies showed that Sal B reduced atherosclerotic lesion area (*P* < 0.05), lipid deposition (*P* < 0.05) and plaque size (*P* < 0.05), lipid levels (TC, TG, LDL) (*P* < 0.05) and inflammatory responses (TNF-α, IL-6, IL-1β) (*P* < 0.05), as well as inhibiting phosphorylation of NF-κB and IκB proteins (*P* < 0.05). Time-dose interval analyses showed that Sal B was relatively effective at doses ranging from 2 to 100 mg/kg, with an intervention period of 4–14 weeks, administered either via gavage or intraperitoneal injection.

**Conclusion:**

Our findings suggest that Sal B effectively delays the progression of atherosclerosis and represents a promising anti-atherosclerotic drug candidate. Further studies are required to translate these promising preclinical findings into the clinical treatment of atherosclerosis.

## 1 Introduction

Atherosclerosis (AS) is the main pathological basis of various cardiovascular diseases and is the main cause of morbidity and mortality worldwide (Tsao et al., 2023). It mainly involves the endothelium of large and medium-sized arteries, and is characterised by lipid deposition and atheromatous plaque formation, which over time leads to thickening of the arterial wall and narrowing of the lumen, causing ischemic changes in the corresponding organs; when plaque rupture occurs, it leads to acute interruption of blood flow in the corresponding organs supplied by the blood, inducing an acute cardiovascular disease (CVD) event (Libby et al., 2019). CVD is estimated to cause more than 18 million deaths annually worldwide. In Europe alone, more than 60 million years of life are lost annually (Townsend et al., 2022). Therefore, timely control of the development of atherosclerosis is important for the prevention of CVD and improvement in the quality of life.

Currently, AS treatment mainly comprises lipid-lowering drugs, anti-inflammatory drugs, antioxidant drugs, and interventional therapies (Wang et al., 2023). Although these therapies have shown relative safety and efficacy in long-term application, several challenges remain. The post-surgical period is accompanied by continuous anticoagulation therapy and the risk of restenosis (Zhang et al., 2024), as well as poor water solubility and non-specific distribution of the drugs, which may lead to adverse effects such as liver injury and rhabdomyolysis, ultimately affecting patient adherence and prognosis (O'Toole et al., 2022; Asare and Georgakis, 2024; Mao et al., 2025). According to data from the National Health and Nutrition Examination Survey (Thompson-Paul et al., 2023), although the 2018 cholesterol guidelines suggest that early identification and control of lipids in patients can reduce the lifetime risk of ASCVD, the proportion of patients taking statins as recommended remains below 50%, resulting in a still suboptimal rate of cardiovascular disease treatment. The reasons for not taking medication were mainly patients’ concerns about side effects and the risk of drug interactions (Ngo-Metzger et al., 2019). Therefore, while optimizing existing therapies, the development of safer and multitarget therapeutic agents remains a key direction in current atherosclerosis research.

Recently, natural active ingredients from medicinal plants and traditional Chinese medicine have been shown to have potential in the treatment of atherosclerosis (Wang et al., 2024). They have the advantages of fewer adverse effects and a multitarget pharmacodynamic approach (Zhang et al., 2023). *Salvia miltiorrhiza*, a plant of the family Labiatae, is one of the oldest traditional Chinese medicines exhibiting efficacy in activating blood circulation and removing blood stasis and has been widely used in the treatment of cardiovascular diseases for thousands of years (Ma et al., 2022). The dried rhizome of *S. miltiorrhiza* is used as the herbal medicine. The main active components of *Salvia divinorum*, which exert medicinal effects, are classified into two categories: water-soluble and fat-soluble components. The most abundant and biologically active water-soluble component is salvianolic acid B (Wei et al., 2023). Salvianolic acid B (Sal B) has been reported by recent preclinical studies to exert protective effects against atherosclerosis through multiple targets, such as lipid reduction, protection of vascular endothelial cells, inhibition of inflammation, and regulation of macrophage activation (Ho and Hong, 2011). Nonetheless, the lack of clinical evidence limits the application of Sal B in the treatment of atherosclerosis. Therefore, a quality assessment and meta-analysis of preclinical studies using Sal B in animal models of atherosclerosis was conducted to assess the effectiveness of Sal B against atherosclerosis in preclinical studies and to explore its mechanisms for future clinical studies and applications.

## 2 Methods

This review was designed and conducted according to the Preferred Reporting Items for Systematic Evaluation and Meta-Analyses (PRISMA) statement. The program was registered in PROSPERO (registration number: CRD42024614887).

### 2.1 Search strategy

A search was conducted on the following eight databases from the time of database establishment to November 2024: PubMed, Embase, Cochrane Library, Web of Science, China Biomedical Literature Service System (Sinomed), China Science and Technology Journal Database (VIP), China Knowledge Network (CNKI), and Wanfang Data. The search terms were based on the search components “Atherosclerosis” and “Salvianolic acid B.” Additionally, references and clinical trials were screened for relevant studies. The complete search strategy is shown in Supplementary Table S1.

### 2.2 Study selection

Studies were included if they met the following criteria: (1) atherosclerotic rat or mouse models with no restriction on modelling methods; (2) treatment groups that could receive Sal B at any dosage, dosing time, or treatment modality; and (3) control groups that received only an equivalent amount of saline or did not receive any treatment. (4) Primary outcome indicators included pathological changes in aortic plaque, lipid levels (TC, TG, HDL, LDL), and inflammatory markers (TNF-α, IL-6, IL-1β). Secondary outcome indicators were protein phosphorylation levels (p-NF-κB, p-IκB) associated with the development of atherosclerosis.

The exclusion criteria were as follows:(1) reviews, conference abstracts, case reports, *in vitro* studies, and clinical trials; (2) treatment groups receiving tanshinic acid complexes or Sal B in combination with other therapies; (3) studies that could not be obtained through reasonable access; (4) absence of predefined outcome metrics; and (5) duplicate data or publications.

### 2.3 Data extraction

The retrieved literature was imported into EndNote 21, and the titles and abstracts of the retrieved studies were independently screened by two evaluators to identify articles that met the above inclusion criteria. Eligibility was determined by reading the full text of eligible studies. When an agreement could not be reached regarding the eligibility of a study, a third reviewer was consulted to resolve the dispute.

The information extracted will include: (1) the first author’s name and year of publication; (2) specific details of the animals in each study, including species, number, sex, weekly age, and body weight; (3) AS modelling method and anesthesia method; (4) Sal B treatment information, including dosage, mode of administration, and duration of treatment, and the corresponding information in the control group; and (5) mean and standard deviation (SD) of the results. If Sal B outcomes were presented for multiple time points or doses in the article, only data for the longest time point and highest dose group were extracted. If the outcome metrics were presented only in graphical form, the authors were contacted in an attempt to obtain the original experimental data. If the authors did not respond, the graphical data were quantified using WebPlotDigitizer 4.5 software (https://automeris.io/WebPlotDigitizer).

### 2.4 Risk-of-bias assessment

Two evaluators assessed the quality of the included animal studies using a 10-item risk-of-bias tool developed by the Systematic Evaluation Center for Animal Laboratories (SYRCLE). The tool assesses criteria for (1) sequence generation; (2) baseline characteristics; (3) covert grouping; (4) animal placement randomisation; (5) blinding (blinding of animal keepers and investigators); (6) assessment of randomisation outcomes; (7) Blind method (result evaluator); (8) Incomplete data report; (9) selective reporting of outcomes; (10) other sources of bias. Any disagreements during the assessment process were resolved by consultation with a third evaluator.

### 2.5 Statistical analyses

Statistical analyses were performed using STATA software version 15.0. The outcome indicators of this study were continuous variable-type data; therefore, standardized mean difference (SMD) and 95% CI were used to indicate effect sizes. Heterogeneity between the studies was assessed using *I* ^2^ statistics. If no heterogeneity between study outcomes was found (*I* ^2^ ≤50%), the meta-analysis was conducted using a fixed-effects model. If heterogeneity between study outcomes was detected (*I* ^2^ >50%), a random-effects model was applied. Statistical significance was set at *P* < 0.05. Subgroup analyses were performed on data with high heterogeneity to explore the sources of heterogeneity. Sensitivity analyses were conducted when individual results showed a significant bias. Potential publication bias for significant indicators was assessed using Egger’s test. If publication bias was present, the trim-and-fill method was employed. To better reveal the influence of Sal B dose and duration, time-dose interval analyses were conducted on aortic atherosclerotic lesion areas, TC, LDL, and TNF-α, which were plotted using Origin 2024.

## 3 Results

### 3.1 Study inclusion

**FIGURE 1:**
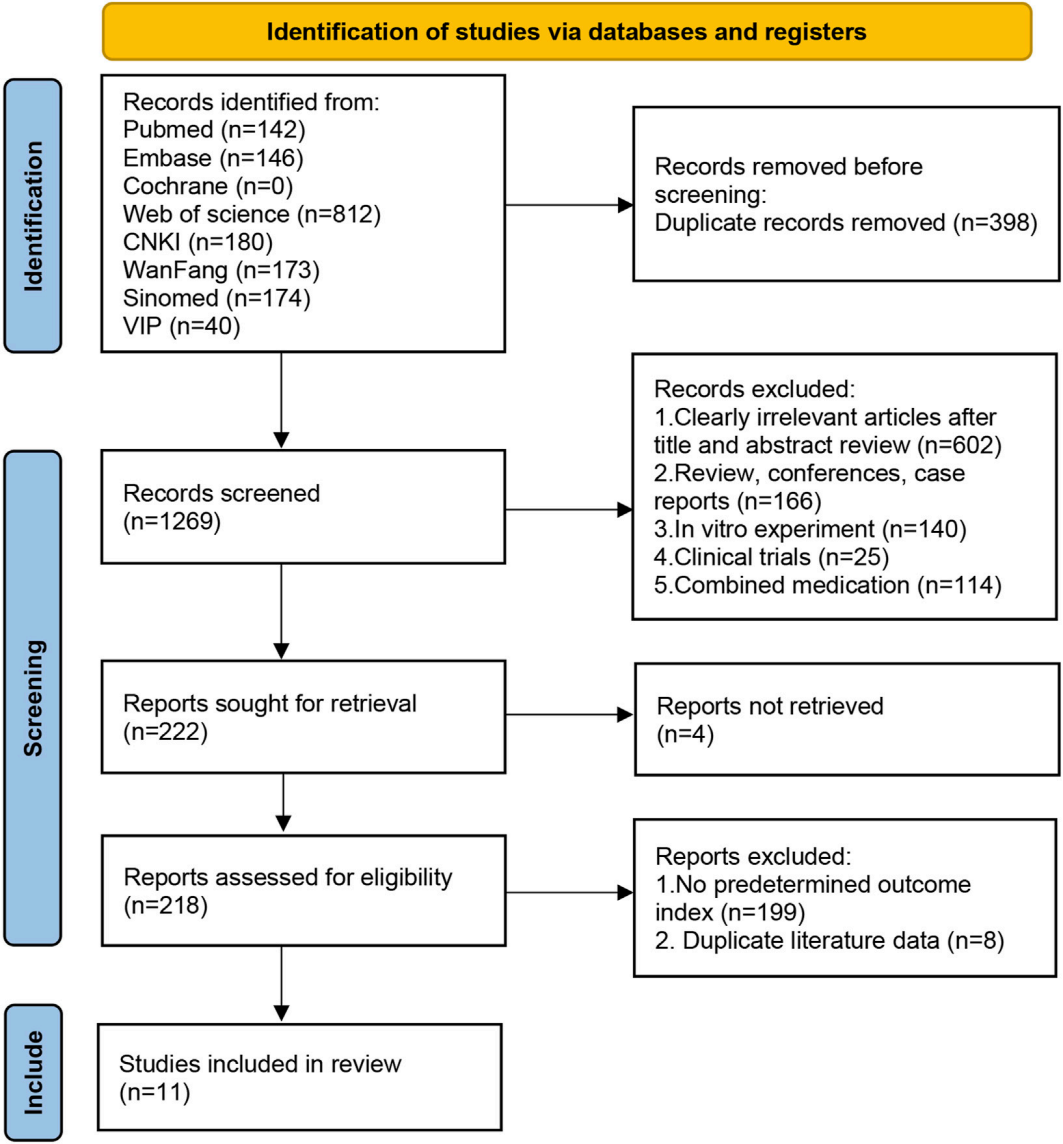
Literature retrieval flow chart.

A total of 1,667 relevant papers were retrieved; after excluding duplicates, 1,269 articles remained. Further reading of the title abstracts excluded irrelevant literature (*n* = 602), reviews, conferences, case reports (*n* = 166), *in vitro* experiments (*n* = 140), clinical trials (*n* = 25), and drug combinations (*n* = 114). After further exclusion of articles that could not be retrieved (*n* = 4), 218 were included in the complete manuscript screening. Finally, 11 articles were included after excluding those without predetermined outcome indicators (*n* = 199) and those with duplicate data (*n* = 8). The screening process is shown in Figure 1.

### 3.2 Characteristics of the included studies

**FIGURE 2:**
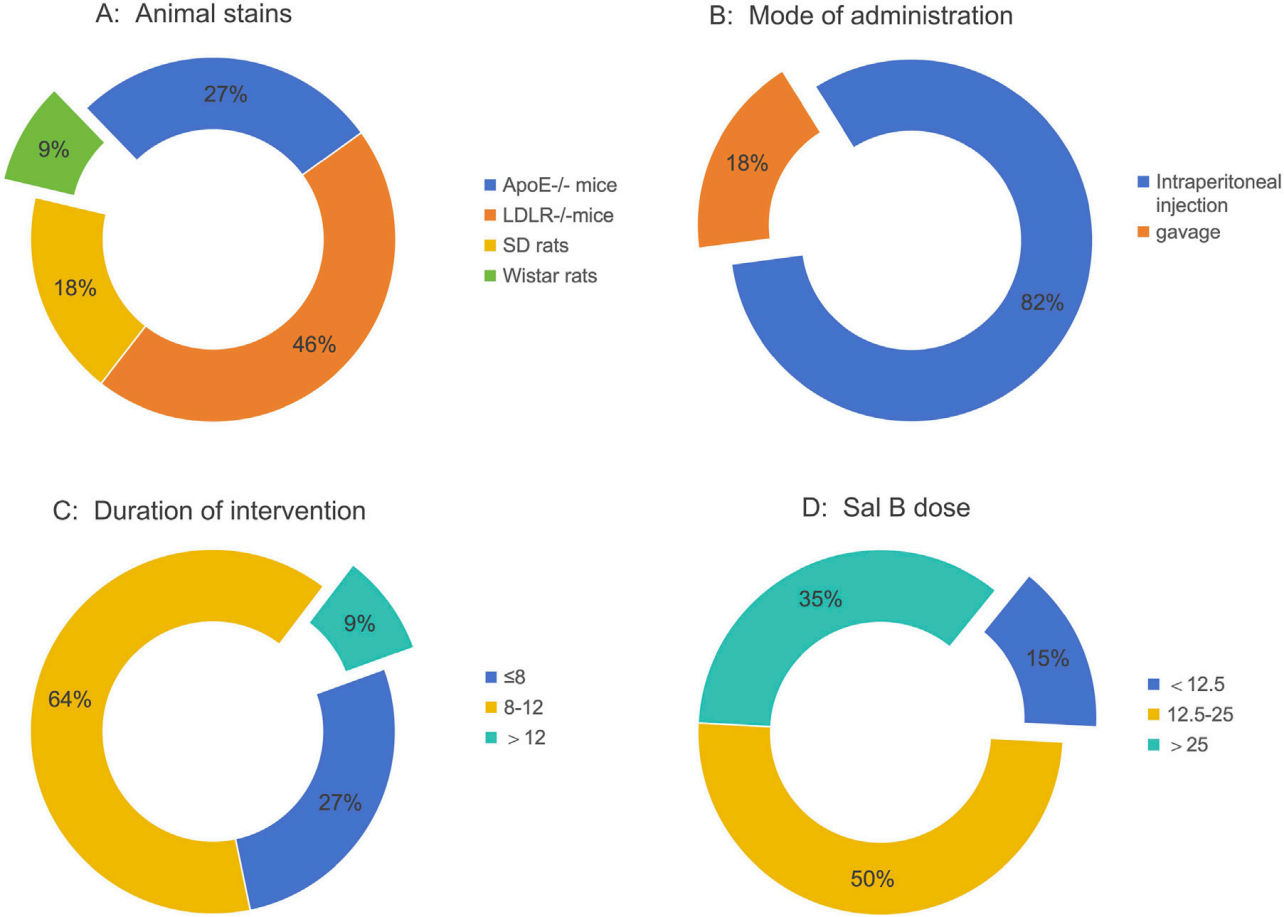
Study characteristics of studies. **(A)** Animal strains, **(B)** Mode of administration, **(C)** Duration of intervention, **(D)** Sal B.

Eleven studies with 275 animal models of atherosclerosis were included. The sample size for each study ranged from 6 to 12 animals. Two studies used Sprague-Dawley rats, one used Wistar rats, three used ApoE^−/−^ mice, and five used LDLR^−/−^ mice (Figure 2A). The animal models used in the included studies were rats or mice. Eleven of the studies involved all male animals. All 11 studies induced HFD in their model constructs for atherosclerosis. Two studies employed gavage, and nine studies used intraperitoneal injection (Figure 2B). The minimum and maximum durations of administration were 4 weeks and 14 weeks, respectively (Figure 2C). The minimum and maximum doses of Sal B were 2 mg/kg/day and 250 mg/kg/day, respectively (Figure 2D). Table 1 summarizes the characteristics of the included studies, whereas Supplementary Table S2 summarizes Sal B used in the included studies.

**TABLE 1:** Characteristics of the included studies.

Study (year)	Model	Species (age, sex, *n* = treatment/model group)	Weight	Diet	Anesthetic	Model group	Treatment group	Duration of intervention	Assessment of atherosclerotic lesion	Outcome index
Chen (2009)	Sprague-Dawley rats	(8 weeks, male, 6/6)	300 g	High-fat diet	NM	NaCl 2 mL/d, gavage	Sal B 9, 18, and 27 mg/kg/d, gavage	6 weeks	Aortic root stained with HE	1. TC, TG, HDL, LDL 2. TNF-α, IL-6
Chi (2010)	Sprague-Dawley rats	(NM, male, 10/10)	130–150 g	High-fat diet	NM	NaCl 2, 12.5, and 20 mg/kg/d, intraperitoneal injection	Sal B 2, 12.5, and 20 mg/kg/d, intraperitoneal injection	8 weeks	NM	1. TC, HDL
Pei (2021)	ApoE^−/−^ mice	(8 weeks, male, 12/12)	19–21 g	High-fat diet	Pentobarbital sodium	0.9% NaCl, intraperitoneal injection	Sal B 30 mg/kg, intraperitoneal injection	4 weeks	Aortic root stained with Oil Red O, HE	1. Atherosclerotic lesion area 2. Oil Red O staining of the aortic root 3. H&E staining of the aortic root 4. TC, TG, HDL, LDL 5. TNF-α, IL-6, IL-1β
Yang (2004)	Wistar rats	(6 weeks, male, 10/10)	250–300 g	High-fat diet	NM	NaCl 25 and 250 mg/kg/d, gavage	Sal B 25 and 250 mg/kg/d, gavage	8 weeks	Aortic root stained with Oil Red O	1. Atherosclerotic lesion area
Zhang et al. (2022b)	LDLR^−/−^ mice	(6 weeks, male, 8/8)	18–23 g	High-fat diet	NM	NaCl 200 μL, intraperitoneal injection	Sal B 25 mg/kg/d, intraperitoneal injection	12 weeks	Aortic root stained with Oil Red O	1. Oil Red O staining of the aortic root 2. TC, TG 3. TNF-α, IL-6, IL-1β 4. p-NF-κB, p-IκB
Zhang and Liu (2023)	LDLR^−/−^ mice	(6 weeks, male, 8/8)	18–23 g	High-fat diet	NM	NaCl 200 μL, intraperitoneal injection	Sal B 25 mg/kg/d, intraperitoneal injection	12 weeks	Aortic root stained with Oil Red O	1. Atherosclerotic lesion area 2. TC, TG, LDL 3. TNF-α, IL-6, IL-1β 4. p-NF-κB, p-IκB
Zhang et al. (2022a)	LDLR^−/−^ mice	(6 weeks, male, 8/8)	18–23 g	High-fat diet	NM	0.9% NaCl 200 μL, intraperitoneal injection	Sal B 25 mg/kg/d, intraperitoneal injection	12 weeks	Aortic root stained with Oil Red O	1. Atherosclerotic lesion area 2. TC, TG, LDL
Zhao et al. (2023)	LDLR^−/−^ mice	(8 weeks, male, 10/10)	26 ± 2 g	High-fat diet	Carbon dioxide	NaCl 12.5, 25, and 50 mg/kg/d, intraperitoneal injection	Sal B 12.5, 25, and 50 mg/kg/d, intraperitoneal injection	14 weeks	Aortic root stained with Oil Red O, HE	1. Atherosclerotic lesion area 2. Oil Red O staining of the aortic root 3. H&E staining of the aortic root 4. TC, TG, LDL
Yang et al. (2020)	ApoE^−/−^ mice	(8 weeks, male, 12/12)	NM	High-fat diet	Pentobarbital sodium	0.9% NaCl, NM, intraperitoneal injection	Sal B 30 mg/kg/d, intraperitoneal injection	4 weeks	Aortic root stained with Oil Red O	1. Atherosclerotic lesion area 2. H&E staining of the aortic root 3. TNF-α, IL-6, IL-1β 4. TC, TG, HDL, LDL
Pan et al. (2022)	LDLR^−/−^ mice	(8 weeks, male, 6/6)	NM	High-fat diet	Pentobarbital sodium	NM	Sal B 30 and 100 mg/kg/d, intraperitoneal injection	8 weeks	Aortic root stained with Oil Red O and HE	1. Atherosclerotic lesion area 2. Oil Red O staining of the aortic root 3. H&E staining of the aortic root
Chang et al. (2024)	ApoE^−/−^ mice	(NM, male, 9/9)	24 g	High-fat diet	Carbon dioxide	NaCl, intraperitoneal injection	Sal B 6.25 and 12.5 mg/kg/d, intraperitoneal injection	12 weeks	Aortic root stained with Oil Red O	1. TC, TG, HDL, LDL 2. TNF-α, IL-6

ApoE^−/−^ mice, apolipoprotein E gene knockout mice; HDL, high-density lipoprotein; LDLR^−/−^ mice, low-density lipoprotein receptor knockout mice; LDL, low-density lipoprotein; IL-6, interleukin-6; IL-1β, interleukin-1β; NM, not mentioned; TC, total cholesterol; TG, triglyceride; TNF-α, tumor necrosis factor-α.

### 3.3 Quality of the included studies

**FIGURE 3:**
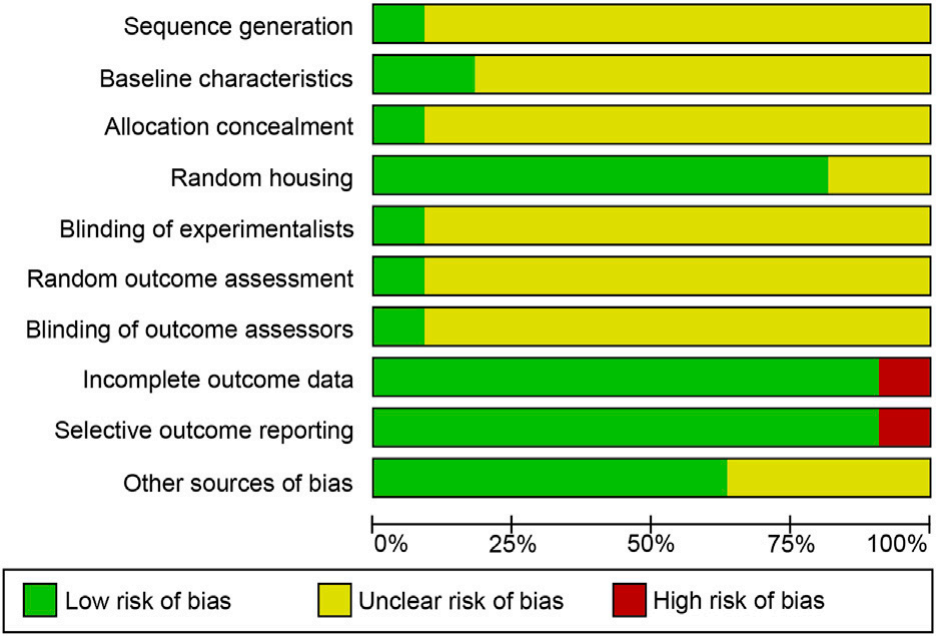
The risk of bias graph of included studies.

The quality of all included studies was assessed in strict accordance with the evaluation criteria. More than half of the criteria were marked as “unclear” because basic information regarding the methodology was missing. Eleven studies mentioned randomized grouping, but only one explicitly used a computerized randomizer for the random allocation methodology and assessment of randomized outcomes. Two studies performed modelling prior to random allocation. One study mentioned a hidden grouping coded by a third party using a random number table. Nine studies considered temperature, humidity, and luminance controls. One study reported the use of a randomization system for randomized grouping, which was coded by a third party through a table of random numbers. Nine studies took temperature, humidity, and brightness controls into account. One study mentioned investigator blinding and the outcome measure. Ten studies had fully reported data. Ten studies clearly reported all outcome indicators. Figure 3 presents the results of the quality assessment for the included studies, and Supplementary Table S3 shows the results of the complete quality assessment.

### 3.4 Outcome measurements

#### 3.4.1 Atherosclerotic lesion area

**FIGURE 4:**
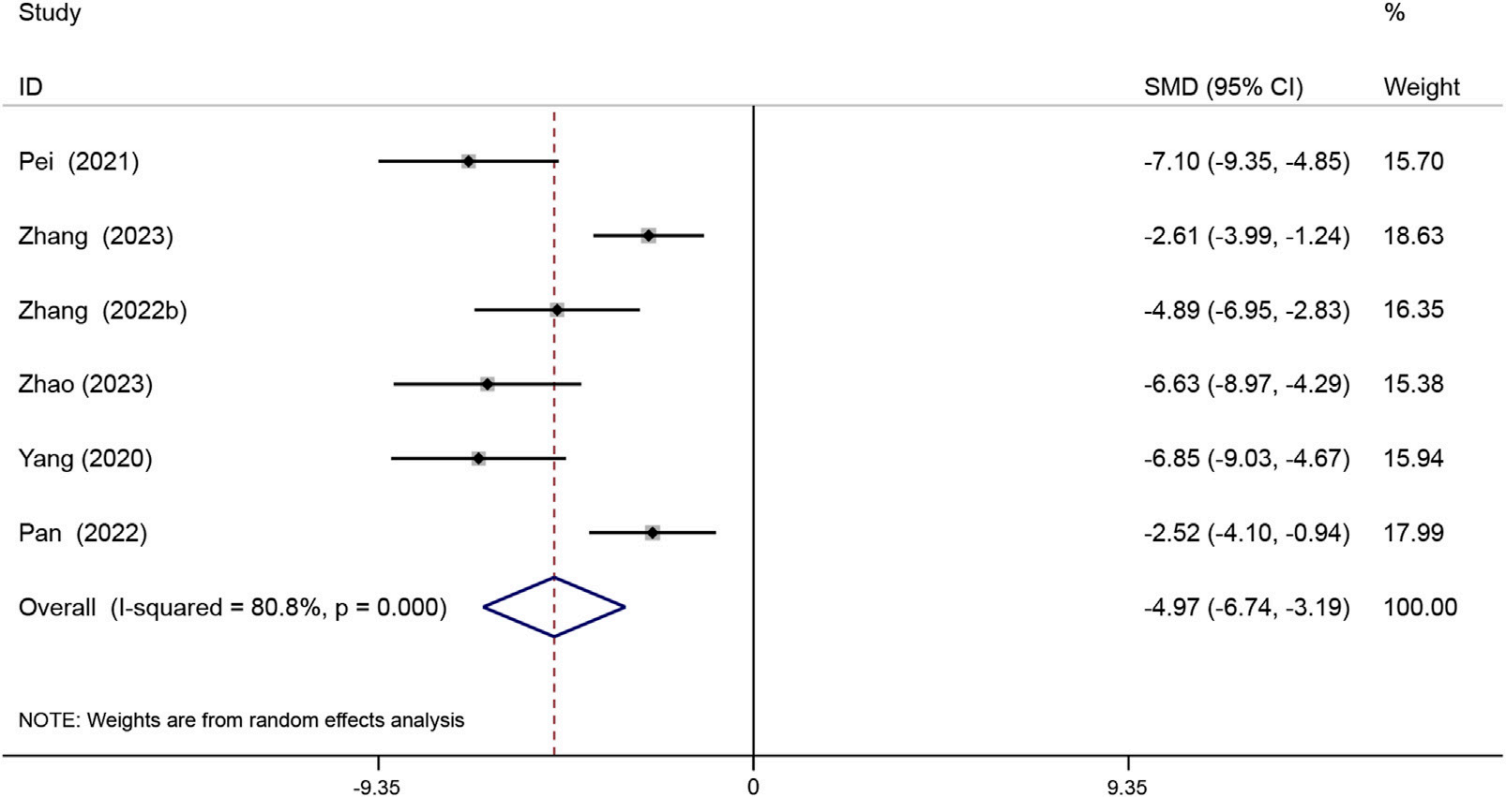
Forest map analyzing the effect of Sal B treatment on aortic sclerosis lesion area.

The atherosclerotic lesion area is expressed as a percentage of the total aortic area covered by the lesion. Six studies reported the aortic atherosclerotic lesion areas. The results showed that Sal B was effective in reducing atherosclerotic lesion area compared with the model group (SMD = −4.97, 95% CI (−6.74, −3.19), *P* < 0.05, *I* ^2^ = 80.8%) (Figure 4).

#### 3.4.2 Lipid deposition and plaque size

**FIGURE 5:**
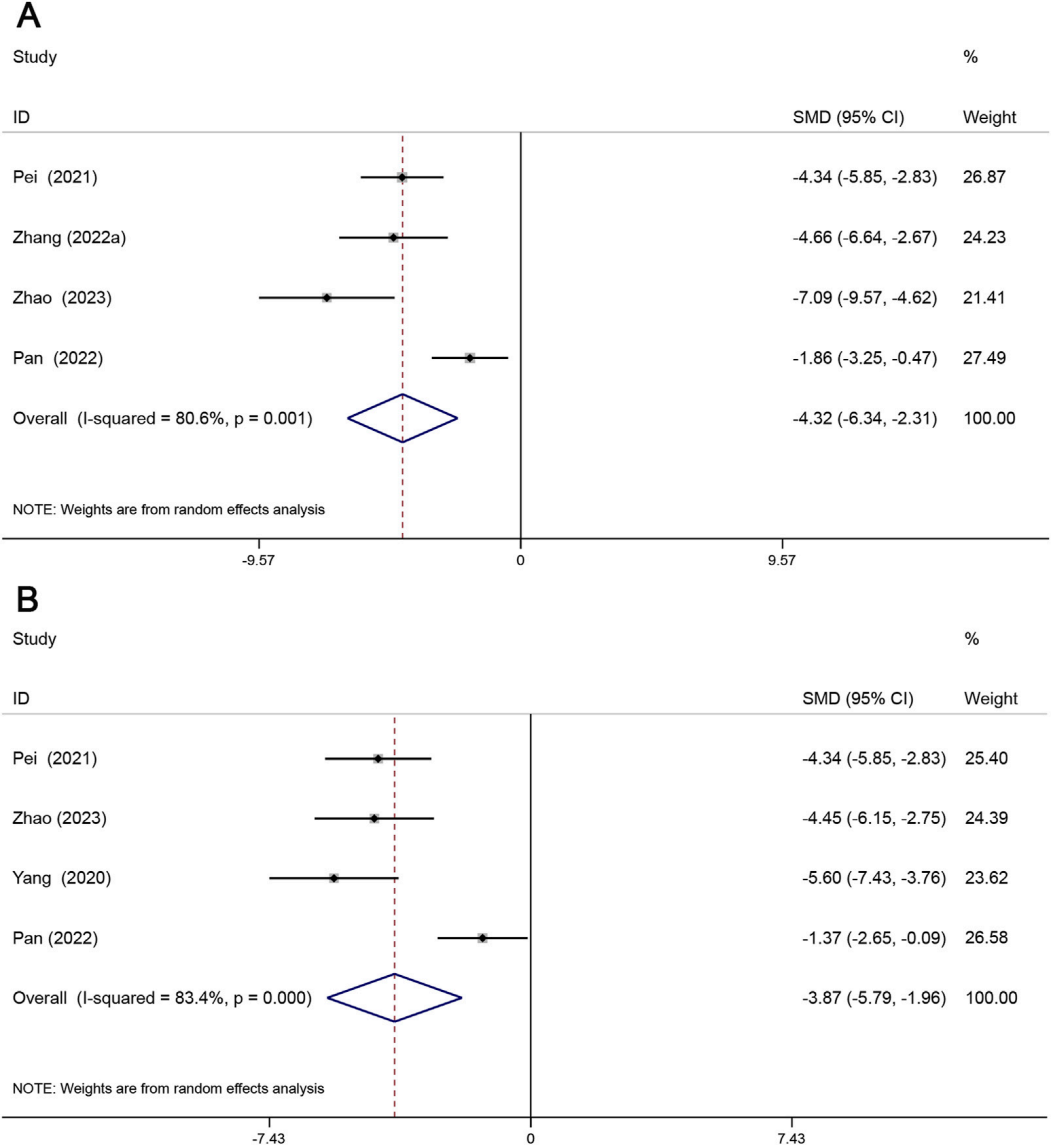
Forest maps show the effects of Sal B on lipid deposition **(A)** and patch size **(B)** in animal models of AS.

Oil red staining was mainly used to detect lipid deposition in the cross-section of the aortic root and was expressed as the percentage of the oil red-stained area to the total area of the aortic root. H&E staining is mainly used to detect the size of atherosclerotic plaques and is expressed as a percentage of the HE-stained area over the total area of the aortic root. Four studies reported the lipid content within the plaque, and four studies reported the plaque size. As shown, lipid content within plaques (SMD = −4.32, 95% CI (−6.34, −2.31), *P* < 0.05, *I* ^2^ = 80.6%) and plaque size (SMD = −3.87, 95% CI (−5.79, −1.96), *P* < 0.05, *I* ^2^ = 83.4%). The results showed that Sal B effectively reduced lipid deposition and plaque size. (Figures 5A, B).

#### 3.4.3 Lipid metabolism

**FIGURE 6:**
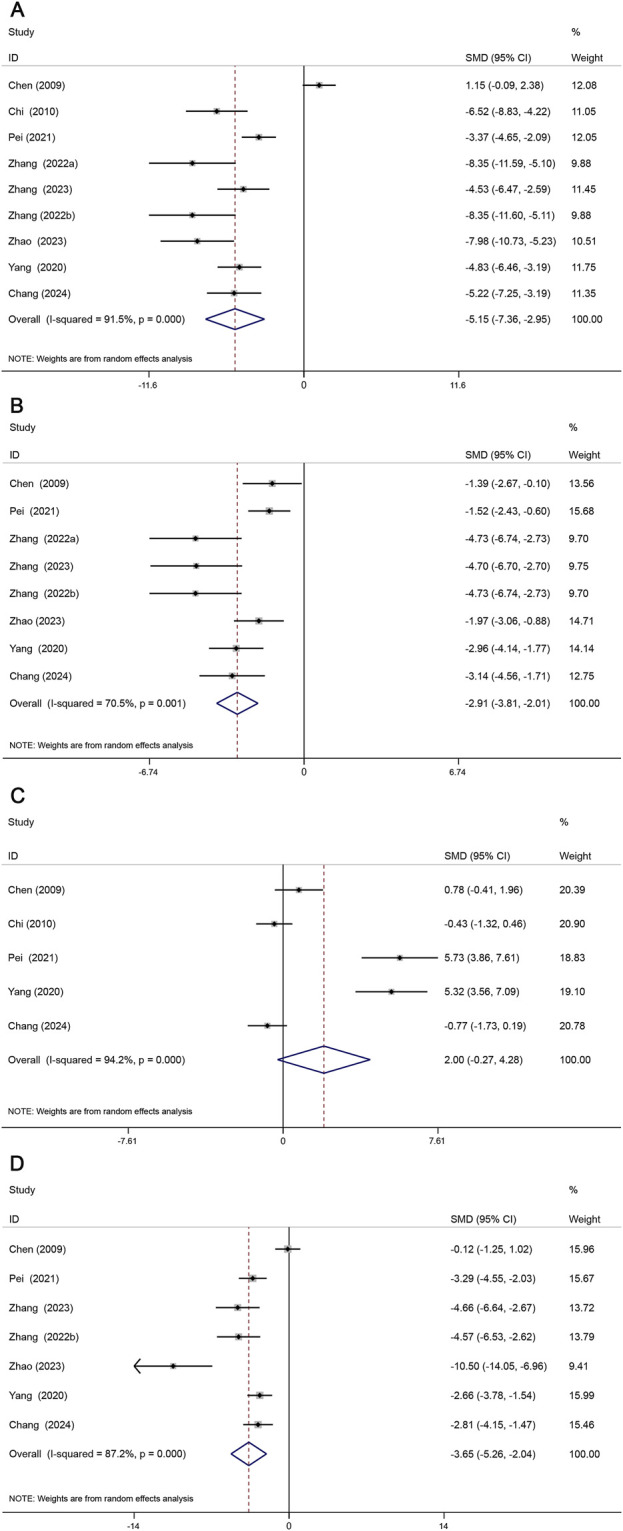
Forest map of the effects of Sal B on TC **(A)**, TG **(B)**, HDL **(C)** and LDL **(D)**.

Lipid metabolism plays a central role in AS (Ding et al., 2017), and the TC, TG, HDL, and LDL indices are of great significance in the assessment of lipid profiles with great significance (Xi et al., 2024). Nine studies assessed TC levels, eight studies assessed TG levels, five studies assessed HDL levels, and seven studies assessed LDL levels. Our meta-analysis showed that TC (SMD = −5.15, 95% CI (−7.36, −2.95), *P* < 0.05, *I* ^2^ = 91.5%) (Figure 6A); TG (SMD = −2.91, 95% CI (−3.81, −2.01), *P* < 0.05, *I* ^2^ = 70.5%) (Figure 6B); HDL (SMD = 2.00, 95% CI (−0.27, 4.28), *P* = 0.084, *I* ^2^ = 94.2%) (Figure 6C); LDL (SMD = −3.65, 95% CI (−5.26, −2.04), *P* < 0.05, *I* ^2^ = 87.2%) (Figure 6D). Overall, Sal B was effective in reducing serum TC, TG, and LDL levels but had no effect on HDL levels.

#### 3.4.4 Inflammatory biomarkers

**FIGURE 7:**
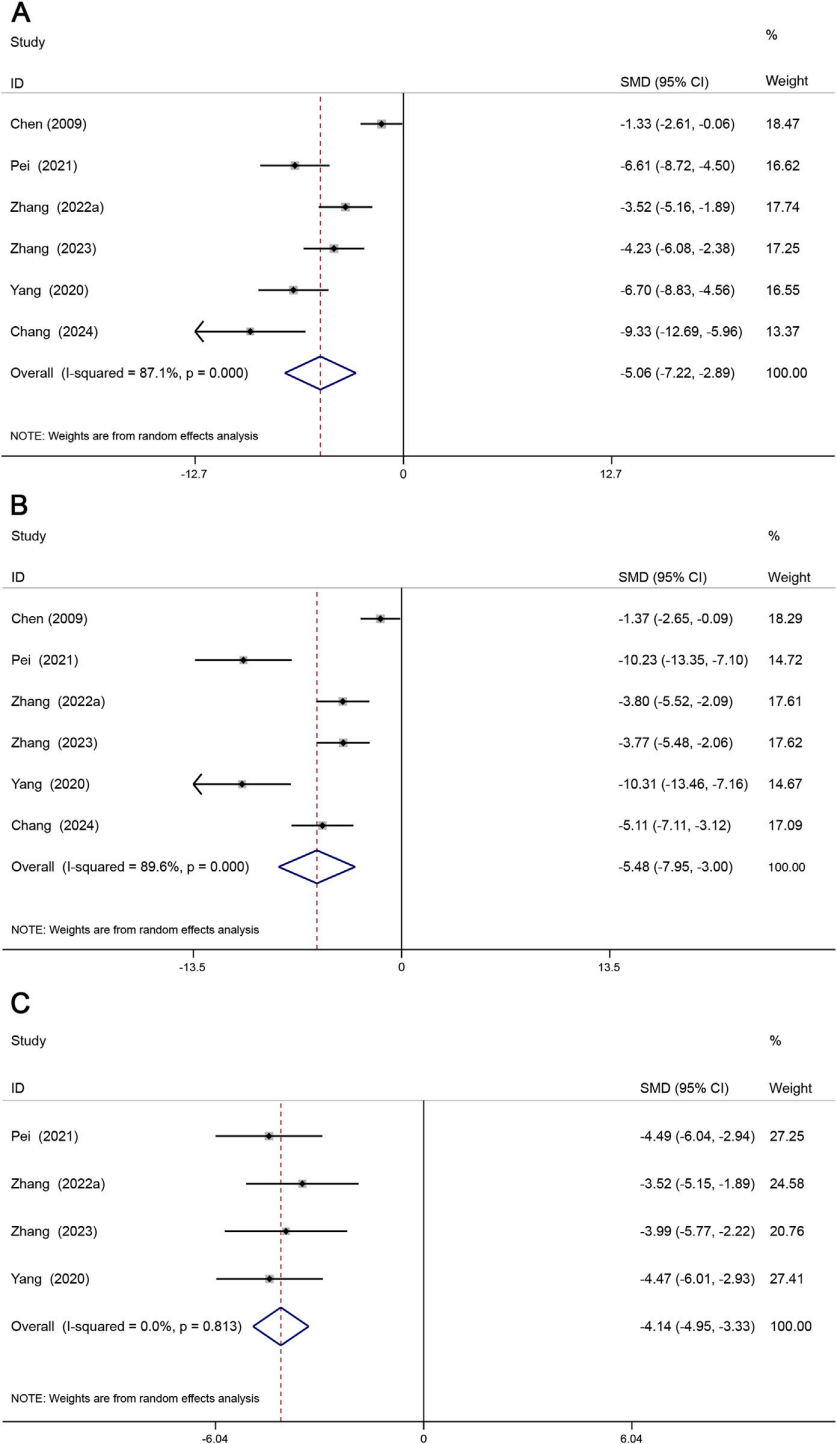
Forest map of the effects of Sal B on TNF-α **(A)**, IL-6 **(B)**, and IL-1β **(C)**.

Inflammatory response plays an important role in the progression of atherosclerosis (Zhu et al., 2018), and TNF-α, IL-6, and IL-1β pro-inflammatory factors tend to induce the development of AS by destroying the vascular endothelium and increasing lipid deposition (Wang and Cui, 2021). A total of 6 studies reported TNF-α, IL-6, and 4 studies reported IL-1β. The results showed that TNF-α (SMD = −5.06, 95% CI (−7.22, −2.89), *P* < 0.05, *I* ^2^ = 87.1%)) (Figure 7A), IL-6 (SMD = −5.48, 95% CI (−7.95, −3.00), *P* < 0.05, *I* ^2^ = 89.6%) (Figure 7B), IL-1β (SMD = −4.14, 95% CI (−4.95, −3.33), *P* < 0.05, *I* ^2^ = 0.0%) (Figure 7C). The results showed that Sal B reduced TNF-α, IL-6, and IL-1β levels compared to the model group.

#### 3.4.5 Inhibition of NF-κB and IκB protein phosphorylation

**FIGURE 8:**
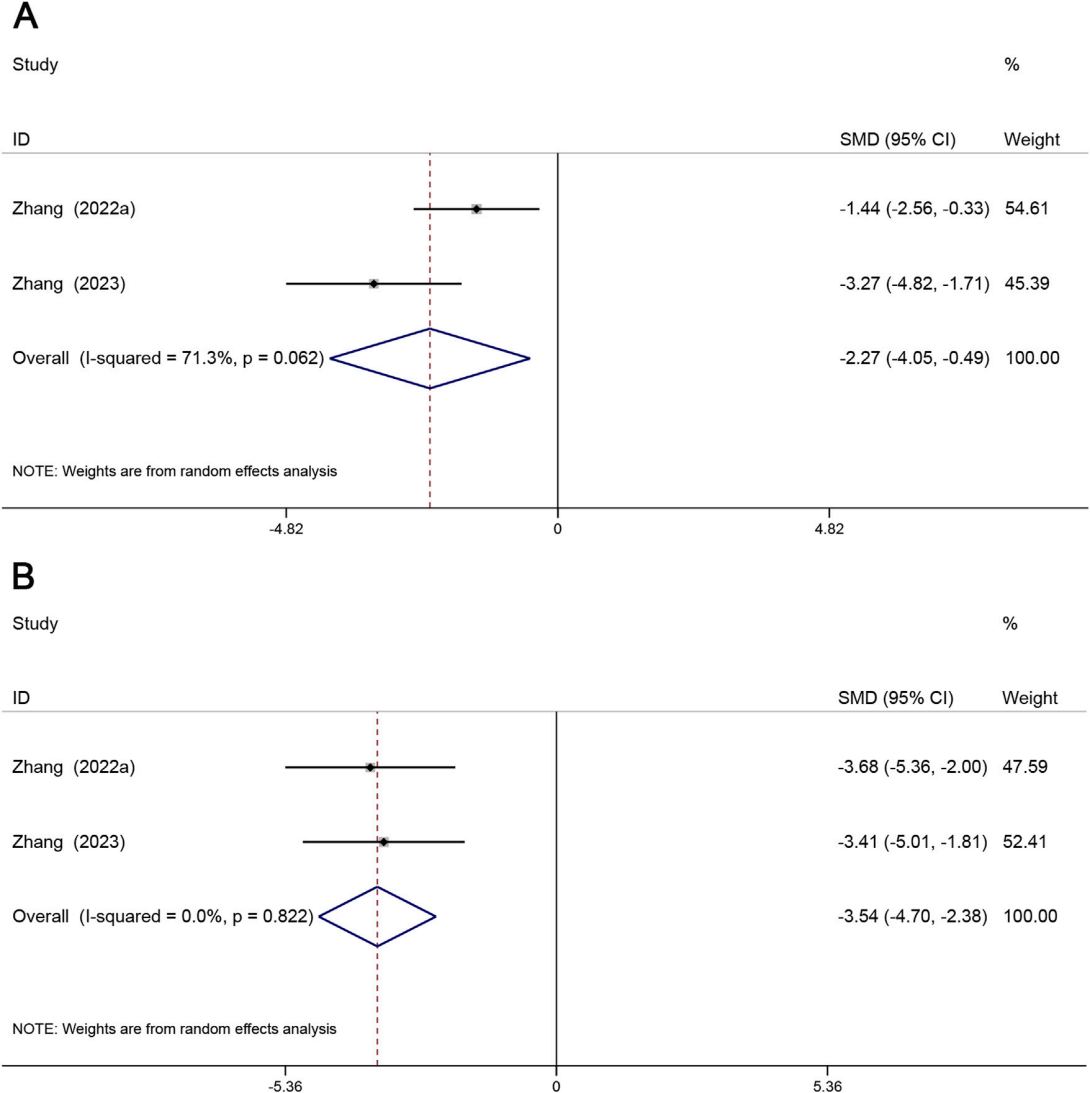
Forest map of effects of Sal B on p-NF-κB **(A)** and p-IκB **(B)**.

Significant activation of NF-κB and IκB is often present in AS tissues, and inhibition of phosphorylated expression of NF-κB and IκB proteins would be important for the prevention of atherosclerosis (Lawrence, 2009). A total of 2 studies reported p-IκB, and 2 studies reported p-NF-κB. p-NF-κB (SMD = −2.27, 95% CI (−4.05, −0.49), *P* < 0.05, *I* ^2^ = 71.3%) (Figure 8A), p-IκB (SMD = −3.54, 95% CI (−4.70, −2.38), *P* < 0.05, *I* ^2^ = 0.0%) (Figure 8B). Compared with the model group, Sal B effectively reduced the phosphorylation levels of NF-κB and IκB.

### 3.5 Subgroup and sensitivity analyses

**FIGURE 9:**
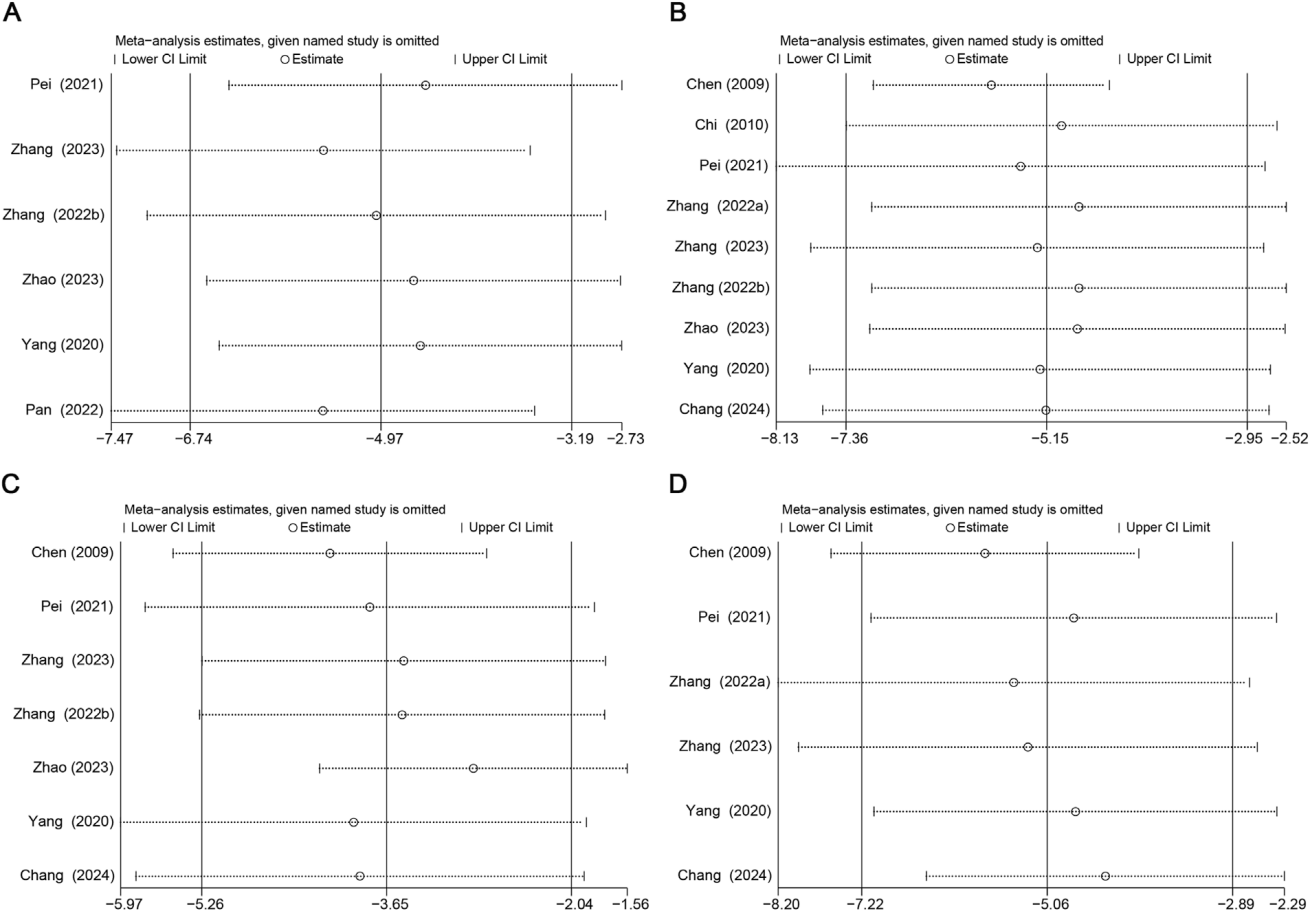
Results of Sensitivity analysis. **(A)** Atherosclerotic Lesion Area, **(B)** TC, **(C)** LDL, **(D)** TNF-α.

Due to high heterogeneity, subgroup analyses of aortic atherosclerotic lesion area, TC, LDL, and TNF-α in terms of duration, Sal B dose, and animal species were performed. The sources of heterogeneity in aortic atherosclerotic lesion regions could be the duration and animal species (Supplementary Table S4). The sources of heterogeneity in TC and LDL could be the duration, Sal B dose, and animal species (Supplementary Tables S5, S6). The heterogeneity in TNF-α may be attributed to differences in Sal B dose and animal species (Supplementary Table S7). Sensitivity analyses for aortic atherosclerotic lesion area, TC, LDL, and TNF-α were performed. After excluding each study from the meta-analysis, no significant effect on the overall effect size was observed (Figure 9).

### 3.6 Publication bias

The publication bias regarding atherosclerotic lesion area, TC, LDL, and TNF-α was evaluated using Egger’s test. The results showed *P* < 0.05, indicating the presence of publication bias (Supplementary Figure S1). The effect of publication bias was further evaluated by applying the trim-and-fill method (Supplementary Figure S2). According to the random-effects model, the results of the trim-and-fill method showed that missing study data did not affect the stability of the results (Supplementary Table S8).

### 3.7 Time-dose interval analysis

**FIGURE 10:**
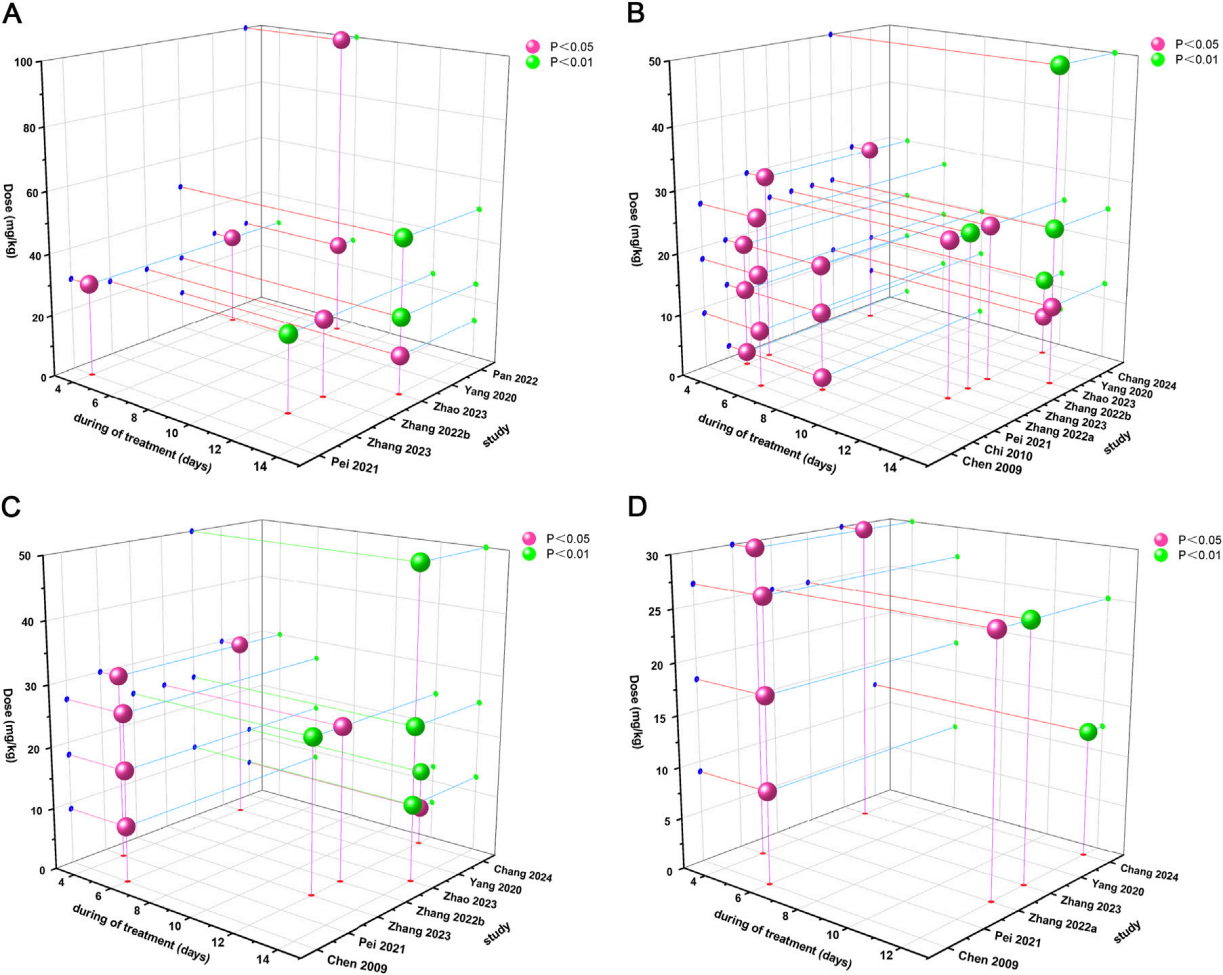
Time-dose interval analysis scatter plot. **(A)** Atherosclerotic Lesion Area, **(B)** TC, **(C)** LDL, **(D)** TNF-α.

Time-dose interval analysis was performed to visualize the optimal drug dose and treatment duration required to achieve optimal efficacy. In order to determine the optimal duration and the most reasonable dose of Sal B for AS, a “time-dose analysis” was conducted based on the actual Sal B dose and treatment period used in the 11 included studies, integrating the results of the effects of aortic atherosclerotic lesion area, TC, LDL, and TNF-α (Figure 10). In the aortic atherosclerotic lesion area analysis, when the Sal B dose was maintained at 12.5–100 mg and the treatment duration was maintained at 4–14 weeks, the improvement effect of Sal B on the aortic atherosclerotic lesion area was significant compared with the model group (*P* < 0.05). In the TC level analysis, when the Sal B dose was maintained at 2–50 mg and the treatment duration was maintained at 4–14 weeks, the improvement effect of Sal B on TC levels was significant (*P* < 0.05) compared to the model group. In the analysis of LDL levels, when the Sal B dose was maintained at 6.25–50 mg and the treatment duration was maintained at 4–14 weeks, the improvement effect of Sal B on LDL levels was significant (*P* < 0.05) compared with the model group. In the analysis of TNF-α level, when the Sal B dose was maintained at 9–30 mg and the treatment duration was maintained at 4–12 weeks, the improvement effect of Sal B on TNF-α level was more significant than that of the model group (*P* < 0.05). The combined results of the time-dose analyses of the different indicators indicated that Sal B demonstrated relatively superior therapeutic effects in the dose range of 2–100 mg/kg and an intervention period of 4–14 weeks.

## 4 Discussion

### 4.1 Summary of efficacy

To the best of our knowledge, this is the first pre-clinical systematic review and meta-analysis to investigate the effects of Sal B on atherosclerosis. Eleven studies involving 275 animals were included in the meta-analysis. In this study, it was found that Sal B reduces atherosclerotic lesion areas, lipid deposition, and plaque size, and may modulate lipid metabolism, reduce inflammatory responses, and inhibit phosphorylation of the associated proteins NF-κB and IκB. In addition, subgroup analyses of atherosclerotic lesion area, TC, LDL, and TNF-α were performed to explore the sources of heterogeneity in terms of time, dose, and model type. The results revealed that the source of heterogeneity in the aortic atherosclerotic lesion area may be the duration and animal species. The sources of heterogeneity in TC and LDL levels may be the duration, Sal B dose, and animal species. The source of heterogeneity in TNF-α may come from Sal B dose versus animal species. Considering the higher heterogeneity, sensitivity analyses for atherosclerotic lesion area, TC, LDL, and TNF-α were performed, which showed that none of the studies had a significant effect on the size of the aggregation effect. In addition, all indicators showed publication bias. The results of the trim-and-fill method showed that missing study data did not affect the stability of the results. The time-dose interval analysis showed that Sal B at doses ranging from 2 to 100 mg/kg with an intervention period of 4–14 weeks was relatively effective.

### 4.2 Possible mechanisms

**FIGURE 11:**
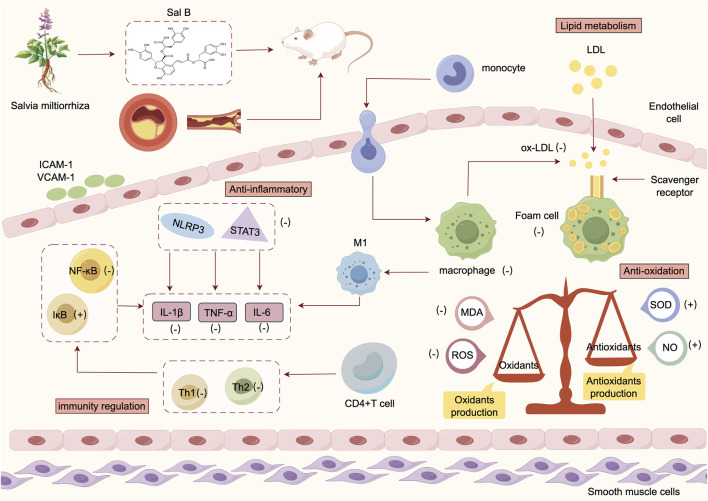
Mechanism of Sal B in the treatment of atherosclerosis.

The results of systematic evaluation of the included studies showed that the protective mechanisms of Sal B against atherosclerosis (Figure 11) mainly consisted of the following:

#### 4.2.1 Lipid metabolism

In lipid metabolism, lipids are deposited in the intima of large- and medium-sized arteries, leading to atherosclerotic plaque formation (Schaftenaar et al., 2016). Lipids in AS lesions originate from the infiltration of plasma lipoproteins, mainly free cholesterol and cholesterol lipids, and, to a lesser extent, triglycerides, phospholipids, and apolipoproteins (Libby et al., 2019). The plasma LDL consists of triglycerides and cholesterol esters, whereas the outer layer consists of phospholipids, free cholesterol, and apolipoprotein B. When endothelial cells are damaged by external stimuli, LDL enters the vasculature and remains in the subendothelium, leading to the accumulation of monocytes in the endothelium where they differentiate into macrophages. Simultaneously, subendothelial LDL is oxidized to ox-LDL. Ox-LDL is a strong ligand for the scavenger receptor CD36, which is easily recognized by macrophages and ingested in large quantities to form foam cells, contributing to AS development (Li et al., 2023). Sal B inhibits macrophage lipid uptake by antagonizing the binding of CD36 to ox-LDL (Wang et al., 2013). LDL is modified through various mechanisms, including cellular oxidation and Cu2+-mediated oxidation (Pryma et al., 2019). Sal B reduces Cu^2+^-induced oxidation of LDL, which in turn reduces ox-LDL-induced endothelial cell damage and high cholesterol-induced neointimal formation (Yang et al., 2011). However, in our meta-analysis, Sal B did not significantly affect HDL levels. Previously, it was believed that the HDL concentration was negatively correlated with the risk of cardiovascular disease (Barter et al., 2007). In contrast, recent studies have shown that elevated HDL concentrations are not always associated with a reduced risk of atherosclerosis, and an improvement in HDL function is more meaningful than the anti-atherosclerotic effect of elevated concentrations (Franczyk et al., 2021).

#### 4.2.2 Inflammatory response

The inflammatory response runs throughout the whole process of AS initiation, progression, and plaque formation. In the early stages of atherosclerosis, endothelial cells are activated and express intercellular adhesion molecule-1 (ICAM-1) and vascular adhesion molecule-1 (VCAM-1), which attract lymphocytes and monocytes to bind and infiltrate the arterial wall, leading to an inflammatory reaction. Thereafter many cells and cytokines are involved in this process, including macrophages, endothelial cells, interleukin (IL), tumor necrosis factor (TNF-α), which further induce apoptosis of smooth muscle cells and promote the progression of atherosclerosis (Zhu et al., 2018). The NLRP3 inflammatory vesicles are cellular sensors that induce inflammation. The NF-κB signalling pathway has been identified as a major molecular mechanism of NLRP3 inflammation. Sal B regulates the NF-κB signalling pathway and inhibits NLRP3 inflammatory vesicle activation to reduce IL-1β secretion and attenuate endothelial damage (Yang et al., 2020). Transcription activator of transcription (STAT3) is an important member of the signal transduction-activating transcription factor family and plays an important role in atherosclerosis by affecting endothelial cell function, macrophage polarization, inflammation, and immunity (Dutzmann et al., 2015). In a study we included (Zhang and Liu, 2023), it was shown that Sal B could exert anti-inflammatory activity by inhibiting the STAT3/NF-κB signalling pathway and suppressing the expression of the inflammatory factors IL-1β, IL-6, and TNF-α, thereby attenuating atherosclerosis.

#### 4.2.3 Immune system

Abnormal immune responses play a crucial role in AS pathogenesis. CD4^+^ T cells are key mediators in the pathogenesis of AS (Chen et al., 2022) and are divided into subtypes such as Th1 and Th2, which are important effector cells in immune responses (Gou and Wang, 2024). A Th1/Th2 imbalance has been suggested as one of the causative mechanisms of atherosclerosis (Wu et al., 2014). Nf-κB is associated with the inflammatory and immune responses of atherosclerosis. Sclerosis inflammatory and immune responses, and can regulate the transcript levels of TNF-α, IL-6, and IL-1β. IκB is an inhibitory protein of NF-κB. TNF-α is an important pro-inflammatory and immunomodulatory factor, and participates in the pathogenesis of AS as a Th1 cytokine. TNF-α activates the phosphorylation of NF-κB and upregulates the activity of NF-κB, and the activation of NF-κB in turn induces the massive expression of inflammatory factors (Chen et al., 2001). Studies have shown that the hallmark of NF-κB/IκB pathway activation is the nuclear translocation of NF-κB (Huang and Guo, 2007). By inhibiting NF-κB/IκB signaling pathway, Sal B can inhibit the phosphorylation of NF-κB and IκB, and reduce the expression of inflammatory factors such as TNF-α, thereby reducing Th1/Th2 imbalance (Zhang and Liu, 2023). Macrophages are involved in all the stages of AS formation. Depending on the environmental stimulus, they are classified as classically activated M1 and alternatively activated M2 types. M1-type macrophages promote the development of AS and produce pro-inflammatory factors such as TNF-α, IL-6, and IL-1β, which exacerbate tissue damage (Mushenkova et al., 2022). Additionally, macrophages phagocytose ox-LDL to form foam cells, and a large number of foam cells are deposited along with vascular smooth muscle cells to form the necrotic core of atherosclerotic plaques (Razeghian-Jahromi et al., 2022). Piezo1 is a Ca^2+^-signalling cation-channel membrane protein that induces cellular excitability and signalling. The MAPK/YAP axis induces Ca^2+^ inward flow, which is closely related to AS development. Studies have shown that Sal B can activate the macrophage Piezo1/MAPK/YAP axis, inhibit foam cell formation and the expression of inflammatory factor proteins, and delay AS formation (Pan et al., 2023).

#### 4.2.4 Oxidative stress

Oxidative stress is closely related to the pathogenesis of atherosclerosis (Shao et al., 2024). An imbalance between free radical production and antioxidant defense systems is the main cause of oxidative stress, which is characterized by the overproduction of ROS and oxidized ox-LDL and is thought to contribute to the progression of atherosclerosis-associated cardiovascular disease (Yan et al., 2024). ROS induces endothelial dysfunction by impairing NO production in endothelial cells, which ultimately contributes to the progression of atherosclerosis. MDA is a lipid peroxidation product produced by peroxidation that is often used as an indicator of the extent of oxidative damage in AS. SOD is a superoxide radical-scavenging factor that maintains the production and inactivation of oxygen radicals and protects cell membranes from damage. NO can affect endothelium-derived contractile factors, inhibit platelet aggregation, and protect endothelial cells. In our included studies, two studies have shown (Zhao et al., 2023; Kong et al., 2022), respectively, that Sal B has a significant endothelial protective effect by inhibiting oxidative stress, decreasing the production of ROS and MDA, and increasing the levels of antioxidant SOD and vasodilator factor NO. Another study (Yang et al., 2020) showed that Sal B inhibited the oxidation of serum LDL, reduced its cytotoxicity, and attenuated atherosclerotic lesions. This suggests that Sal B has a strong antioxidant effect on atherosclerosis, and this mechanism may be related to the maintenance of redox balance and alleviation of lipid peroxidation.

### 4.3 Limitations

(1) Only English and Chinese studies were included in the search, and studies written in other languages were not retrieved. *Salvia divinorum* is a commonly used drug in Asia, which may result in selective bias to some extent.
(2) Data in some studies were not provided directly as raw data but were obtained indirectly through data extraction tools, which may lead to quantitative measurement bias.
(3) Due to the limited literature included, there were limitations in performing subgroup analyses on factors such as sex of experimental animals, modelling, and method of drug administration; the high degree of heterogeneity may also be related to the husbandry environment and sample characteristics.
(4) There were limitations to the methodological quality of the included articles, mainly in the form of potential bias, as most studies did not report in detail the specific methods used for random allocation and blinded implementation. It is recommended that future studies report strict adherence to the ARRIVE guidelines.

### 4.4 Implications

Systematic reviews of animal studies play a critical role in bridging the gap between basic preclinical experiments and clinical application (Bahadoran et al., 2020). By systematically integrating and evaluating therapeutic outcomes across different animal models, dosages, and intervention strategies, they provide a more robust evidence base for clinical trial design and enhance the translational relevance of animal research findings.

To minimize the heterogeneity caused by physiological and immunological differences across species, the present study limited the inclusion criteria to rodent models (mice and rats), excluding studies involving rabbits and other species. Nevertheless, it is important to acknowledge that interspecies differences in genetics, drug metabolism, and pathophysiological responses may lead to discrepancies between experimental results in animals and clinical outcomes in humans (Lal et al., 2016). Therefore, the protective effects of Sal B against atherosclerosis observed in animal models should be interpreted with caution.

Defining a safe and effective dose range is crucial for both future animal and clinical research. Among the 11 included studies, none reported adverse effects. Preclinical evidence indicates that intravenous administration of Sal B at doses ≤480 mg/kg was non-toxic in pregnant rats (Cai et al., 2019). A clinical trial also reported good safety and tolerability following a single intravenous injection of 300 mg or repeated daily injections of 250 mg for five consecutive days (Cheng et al., 2023). These findings collectively support the safety of Sal B at current dosage levels.

Time–dose interval analysis in this study showed that Sal B was relatively effective at doses ranging from 2 to 100 mg/kg, with an intervention period of 4–14 weeks, although a considerable variation was observed. This variability may be attributed to differences in sensitivity of animal models to lipid metabolism disturbances and inflammatory responses, which can influence drug efficacy (Yau et al., 2024; Torikai et al., 2023). In addition, variations in administration routes and dosage forms may affect *in vivo* absorption and distribution (Turner et al., 2011).

Therefore, future animal studies should be conducted using standardized and consistent models, with refined gradients of dosage and intervention duration, and accompanied by systematic toxicological evaluations to determine the minimum effective dose, optimal therapeutic window, safety thresholds under different routes of administration, and appropriate delivery strategies for Sal B. These efforts will enhance the overall quality of preclinical evidence and facilitate the clinical translation of Sal B.

## 5 Conclusion

Our findings indicate that Sal B exerts protective effects against atherosclerosis by significantly attenuating disease progression, reducing lipid accumulation, and lowering inflammatory cytokine levels. Its potential mechanisms involve coordinated multitarget effects, including regulation of lipid metabolism, modulation of the immune system, anti-inflammatory activity, and antioxidant properties. Within a dosage range of 2–100 mg/kg and an intervention period of 4–14 weeks, Sal B demonstrated favorable efficacy, suggesting a broad therapeutic window. This study provides important evidence supporting further investigation into the mechanisms and translational potential of Sal B. However, more comprehensive studies are warranted to fully elucidate its long-term safety and therapeutic value.

## Data Availability

The original contributions presented in the study are included in the article/Supplementary Material, further inquiries can be directed to the corresponding author.
