# Supplementary material for: Protective effect of Salvianolic acid B against atherosclerosis: a preclinical systematic review and meta-analysis
Source: Front Pharmacol. 2025 Jun 18;16:1548811. doi: 10.3389/fphar.2025.1548811 (PMC12213384; doi:10.3389/fphar.2025.1548811)

Supplementary Material

**Supplementary Table 1:** Search strategy.

| **Database** | **Search strategy** | **Count** |
| --- | --- | --- |
| PubMed | (("Atherosclerosis"[Mesh]) OR ("atherosclerosis"[Title/Abstract] OR "Atheroscleroses"[Title/Abstract] OR "Atherogenesis"[Title/Abstract] OR "Atherogeneses"[Title/Abstract] OR "carotid atherosclerosis"[Title/Abstract] OR "coronary atherosclerosis"[Title/Abstract] OR "AS"[Title/Abstract] OR "atherosclerotic plaque"[Title/Abstract] OR "atherosclerotic heart disease"[Title/Abstract] OR "coronary atherosclerotic heart disease"[Title/Abstract])) AND (("salvianolic acid" [Supplementary Concept]) OR ("Salvianolic acid"[Title/Abstract] OR "Salvianic acid"[Title/Abstract])) | 142 |
| Embase | #1: 'atherosclerosis'/exp OR 'atherosclerosis'  #2: 'atheriosclerotic disease':ab,ti OR 'athero sclerosis':ab,ti OR 'atheromatous sclerosis':ab,ti OR 'atherosclerosis grading':ab,ti OR 'atherosclerotic disease':ab,ti OR 'atherosclerotic disorder':ab,ti OR 'atherosclerotic vascular disease':ab,ti OR 'atherosclerotic vascular disorder':ab,ti OR atherosclerosis:ab,ti  #3: #1 OR #2  #4: 'salvianolic acid'/exp OR 'salvianolic acid'  #5: 'salvianic acid':ab,ti  #6: #4 OR #5  #7: #3 AND #6 | 146 |
| Web of Science | TS = (atherosclerosis OR Atheroscleroses OR Atherogenesis OR Atherogeneses OR "carotid atherosclerosis" OR "coronary atherosclerosis" OR AS OR "atherosclerotic plaque" OR "atherosclerotic heart disease" OR "coronary atherosclerotic heart disease") AND TS = ("Salvianolic acid B" OR "Salvianic acid B") | 812 |
| Cochrane Library | #1: MeSH descriptor: [Atherosclerosis] explode all trees  #2: Atherosclerosis OR Atherogeneses OR Atherogenesis OR Atheroscleroses  #3: #1 OR #2  #4: 'Salvianolic acid'/exp OR 'Salvianolic acid'  #5: #3 AND #4 | 0 |
| CNKI, Wanfang, VIP, Sinomed | TKA = (动脉粥样硬化 + 动脉硬化 + 动脉闭塞性疾病) AND TKA = (丹酚酸 + 丹参多酚酸 + 丹参丹酚酸) | CNKI (180), Wanfang (173), VIP (40), Sinomed (174) |

**Supplementary Table 2:** Information on Sal B in each study.

| **Study** | **Purity** | **Formulations** | **Source** | **Route of administration** |
| --- | --- | --- | --- | --- |
| Chen 2009 | NM | Solutions (0.9% NaCl) | Chinese Academy of Sciences (CAS) | Gavage |
| Chi 2010 | 98% | Solutions (0.9% NaCl) | Dalian University of Technology Laboratory | Intraperitoneal injection |
| Pei 2021 | NM | Solutions (0.9% NaCl) | Dingrui Chemical (Shanghai) Co. | Intraperitoneal injection |
| Yang 2004 | ≥ 98% | Solutions (0.9% NaCl) | Laboratory, Second Military Medical University | Gavage |
| Zhang 2022a | 98% | Solutions (0.9% NaCl) | Chengdu Pfizer Biotechnology Co. | Intraperitoneal injection |
| Zhang 2023 | 98% | Solutions (0.9% NaCl) | Chengdu Pfizer Biotechnology Co. | Intraperitoneal injection |
| Zhang 2022b | 98% | Solutions (0.9% NaCl) | Chengdu Pfizer Biotechnology Co. | Intraperitoneal injection |
| Zhao 2023 | ≥ 98% | Solutions (0.9% NaCl) | Chengdu Alpha Biotechnology Co. | Intraperitoneal injection |
| Yang 2020 | NM | Solutions (0.9% NaCl) | Dalian Meilun Biotechnology | Intraperitoneal injection |
| Pan 2022 | > 98% | Solutions (DMSO) | Shanghai Selleck Chemical Company | Intraperitoneal injection |
| Chang 2024 | > 94% | Solutions (0.9% NaCl) | Sigma-Aldrich, St. Louis, USA | Intraperitoneal injection |

**Supplementary Table 3:** Methodological quality of the included studies.

| **Study** | **1** | **2** | **3** | **4** | **5** | **6** | **7** | **8** | **9** | **10** |
| --- | --- | --- | --- | --- | --- | --- | --- | --- | --- | --- |
| Chen 2009 | ? | ? | ? | + | ? | ? | ? | - | + | + |
| Chi 2010 | ? | ? | ? | ? | ? | ? | ? | + | - | + |
| Pei 2021 | ? | ? | ? | + | ? | ? | ? | + | + | + |
| Yang 2004 | ? | ? | ? | + | ? | ? | ? | + | + | + |
| Zhang 2022a | ? | ? | ? | + | ? | ? | ? | + | + | ? |
| Zhang 2023 | ? | ? | ? | + | ? | ? | ? | + | + | ? |
| Zhang 2022b | ? | ? | ? | + | ? | ? | ? | + | + | ? |
| Zhao 2023 | ? | + | ? | + | ? | ? | ? | + | + | + |
| Yang 2020 | ? | ? | ? | ? | ? | ? | ? | + | + | + |
| Pan 2022 | ? | + | ? | + | ? | ? | ? | + | + | ? |
| Chang 2024 | + | ? | + | + | + | + | + | + | + | + |

**Supplementary Table 4:** Subgroup analyses of the atherosclerotic lesion area.

| **Parameter** | **Subgroup** | |  | **No. of studies** | **SMD [95% CI]** | ***I*^2^ (%)** |
| --- | --- | --- | --- | --- | --- | --- |
| Atherosclerotic lesion area | Time | ≤ 6 | | 2 | -6.97 [-8.54, -5.41] | 0 |
|  |  | 8–12 | | 3 | -3.17 [-4.49, -1.84] | 48.7 |
|  |  | > 12 | | 1 | -6.63 [-8.97, -4.29] | - |
|  | Dosage | ≤ 25 | | 2 | -3.62 [-5.84, -1.40] | 69.2 |
|  |  | > 25 | | 4 | -5.69 [-8.17, -3.20] | 82.8 |
|  | Species | ApoE^−/−^ mice | | 2 | -6.97 [-8.54, -5.41] | 0 |
|  |  | LDLR^−/−^ mice | | 4 | -3.98 [-5.75, -2.21] | 74.4 |

**Supplementary Table 5:** Subgroup analyses of TC.

| **Parameter** | **Subgroup** |  | | **No. of studies** | **SMD [95% CI]** | ***I*^2^ (%)** |
| --- | --- | --- | --- | --- | --- | --- |
| TC | Time | ≤ 6 | 3 | | -2.33 [-5.89, 1.24] | 95.1 |
|  |  | 8–12 | 5 | | -6.20 [-7.65, -4.76] | 42.7 |
|  |  | > 12 | 1 | | -7.98 [-10.73, -5.23] | - |
|  | Dosage | ≤ 25 | 5 | | -6.20 [-7.65, -4.76] | 42.7 |
|  |  | > 25 | 4 | | -3.63 [-7.09, -0.16] | 94.8 |
|  | Species | SD rats | 2 | | -2.62 [-10.14, 4.89] | 97 |
|  |  | ApoE^−/−^ mice | 3 | | -4.29 [-5.46, -3.12] | 36.6 |
|  |  | LDLR^−/−^ mice | 4 | | -7.05 [-9.22, -4.88] | 60.3 |

**Supplementary Table 6:** Subgroup analyses of LDL.

| **Parameter** | **Subgroup** |  | **No. of studies** | **SMD [95% CI]** | ***I*^2^ (%)** |
| --- | --- | --- | --- | --- | --- |
| LDL | Time | ≤ 6 | 3 | -2.01 [-3.92, -0.10] | 87.5 |
|  |  | 8–12 | 3 | -3.83 [-5.12, -2.54] | 40.1 |
|  |  | > 12 | 1 | -10.50 [-14.05, -6.96] | - |
|  | Dosage | ≤ 25 | 3 | -3.83 [-5.12, -2.54] | 40.1 |
|  |  | > 25 | 4 | -3.58 [-6.11, -1.05] | 92.0 |
|  | Species | SD rats | 1 | -0.12 [-1.25, 1.02] | - |
|  |  | ApoE^−/−^ mice | 3 | -2.90 [-3.61, -2.19] | 0 |
|  |  | LDLR^−/−^ mice | 3 | -6.20 [-9.15, -3.24] | 78.3 |

**Supplementary Table 7:** Subgroup analyses of TNF-α.

| **Parameter** | **Subgroup** |  | **No. of studies** | **SMD [95% CI]** | ***I*^2^ (%)** |
| --- | --- | --- | --- | --- | --- |
| TNF-α | Time | ≤ 6 | 3 | -4.80 [-8.73, -0.88] | 92.9 |
|  |  | 8–12 | 3 | -5.29 [-7.98, -2.60] | 78.7 |
|  | Dosage | < 20 | 1 | -9.33 [-12.69, -5.96] | - |
|  |  | 20–25 | 2 | -3.83 [-5.06, -2.61] | 0 |
|  |  | > 25 | 3 | -4.80 [-8.73, -0.88] | 92.9 |
|  | Species | SD rats | 1 | -1.33 [-2.61, -0.06] | - |
|  |  | ApoE^−/−^ mice | 3 | -7.10 [-8.48, -5.72] | 1.4 |
|  |  | LDLR^−/−^ mice | 2 | -3.83 [-5.06, -2.61] | 0 |

**Supplementary Table 8:** Results from Egger’s test and trim-and-fill analysis.

| **Outcomes** | **Egger’s test *P* value** | **Before the trim-and-fill method** | | | **After the trim-and-fill method** | | |
| --- | --- | --- | --- | --- | --- | --- | --- |
|  |  | ***P* value** | **SMD (random)** | **Number of studies** | ***P* value** | **SMD (random)** | **Number of studies** |
| Atherosclerotic lesion area | *P* = 0.003 | 0.000 | -6.738 | 6 | 0.000 | 0.007 | 6 |
| TC | *P* = 0.006 | 0.000 | -5.155 | 9 | 0.000 | 0.006 | 9 |
| LDL | *P* = 0.018 | 0.000 | -3.652 | 7 | 0.000 | 0.026 | 7 |
| TNF-α | *P* = 0.003 | 0.000 | -5.055 | 6 | 0.000 | 0.006 | 6 |

**Supplementary Figure 1:** Egger’s publication bias plot for (A) Atherosclerotic Lesion Area (B) TC (C) LDL (D) TNF-α.

**
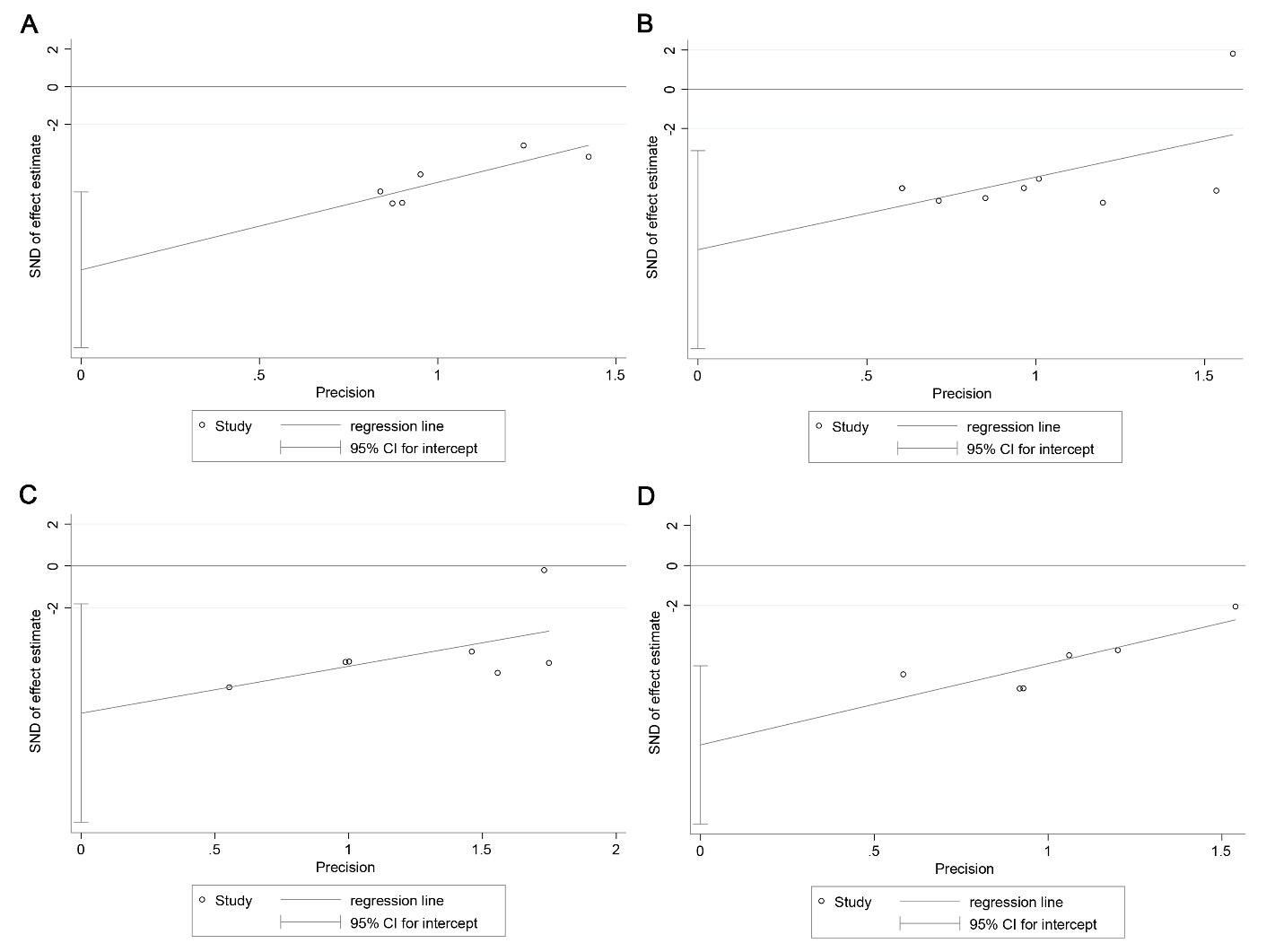
**

**Supplementary Figure 2:** Results of trim and fill method. (A) Atherosclerotic Lesion Area (B) TC (C) LDL (D) TNF-α.


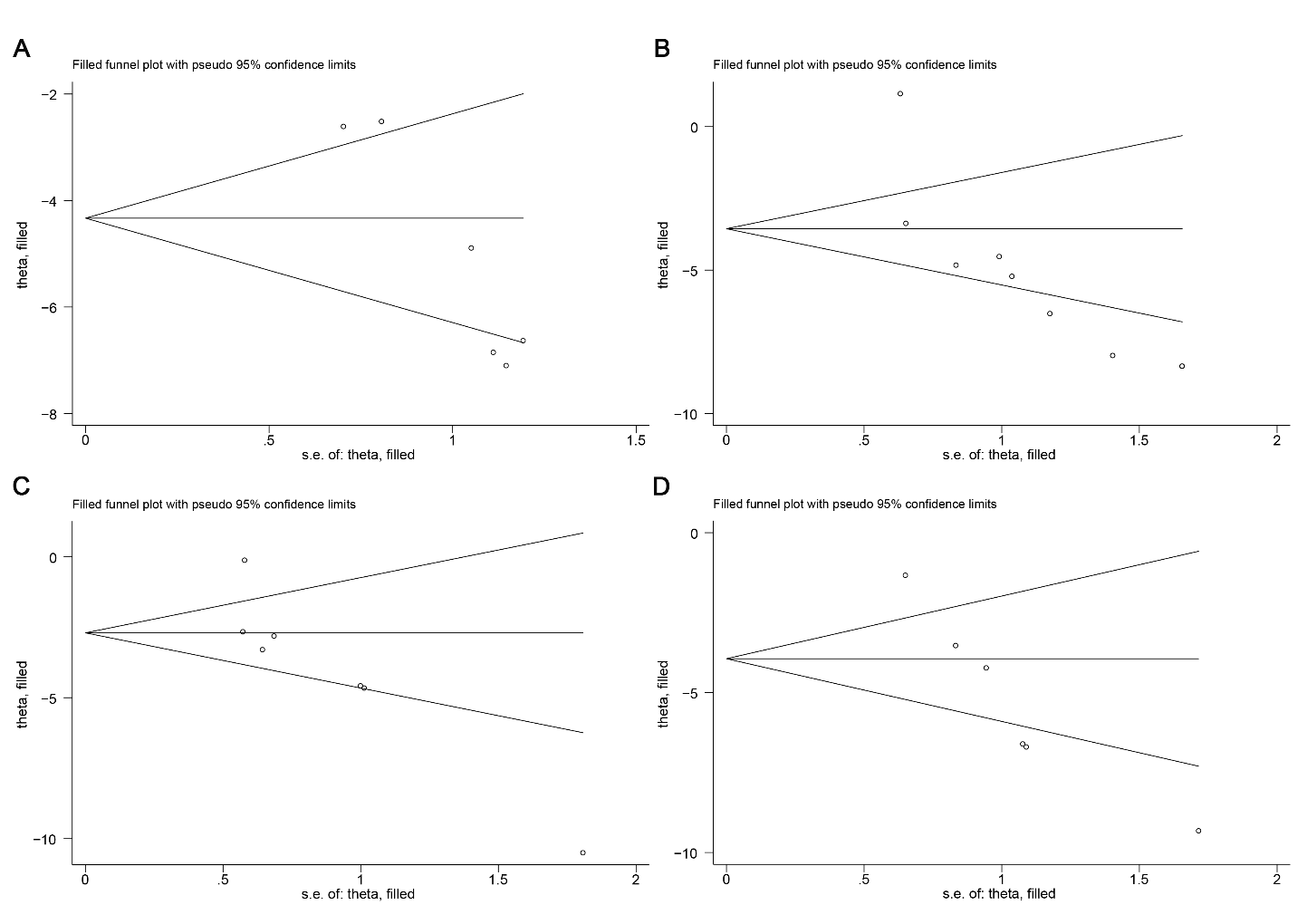

Supplement: Supplementary file 1 [file Supplementaryfile1.docx]
